# The Effect of Plant Proteins Derived from Cereals and Legumes on Heme Iron Absorption

**DOI:** 10.3390/nu7115446

**Published:** 2015-10-30

**Authors:** Valerie Weinborn, Fernando Pizarro, Manuel Olivares, Alex Brito, Miguel Arredondo, Sebastián Flores, Carolina Valenzuela

**Affiliations:** 1 Department of Food Science and Technology, University of California, Davis. 1 Shields Avenue, University of California, Davis, CA 95616, USA; vweinborn@ucdavis.edu; 2 Micronutrients Laboratory, Institute of Nutrition and Food Technology (INTA), University of Chile, Avda. El Líbano 5524, Casilla 13811, Santiago, Chile; fpizarro@inta.uchile.cl (F.P.); molivare@inta.uchile.cl (M.O.); marredon@inta.uchile.cl (M.A.); seb.flores@postgrad.otago.ac.nz (S.F.); 3 United States Department of Agriculture, Western Human Nutrition Research Center, University of California, 430 W. Health Sciences Drive, Davis, CA 95616, USA; abrito@ucdavis.edu; 4 Faculty of Veterinary and Animal Sciences, University of Chile (FAVET), Santa Rosa 11.735, La Pintana, Casilla 2, La Granja, Santiago, Chile

**Keywords:** heme iron, plant protein, iron absorption, human

## Abstract

The aim of this study is to determine the effect of proteins from cereals and legumes on heme iron (Fe) absorption. The absorption of heme Fe without its native globin was measured. Thirty adult females participated in two experimental studies (15 per study). Study I focused on the effects of cereal proteins (zein, gliadin and glutelin) and study II on the effects of legume proteins (soy, pea and lentil) on heme Fe absorption. When heme was given alone (as a control), study I and II yielded 6.2% and 11.0% heme absorption (*p* > 0.05). In study I, heme Fe absorption was 7.2%, 7.5% and 5.9% when zein, gliadin and glutelin were added, respectively. From this, it was concluded that cereal proteins did not affect heme Fe absorption. In study II, heme Fe absorption was 7.3%, 8.1% and 9.1% with the addition of soy, pea and lentil proteins, respectively. Only soy proteins decreased heme Fe absorption (*p* < 0.05). These results suggest that with the exception of soy proteins, which decreased absorption, proteins derived from cereals and legumes do not affect heme Fe absorption.

## 1. Introduction

Affecting over 1.62 billion people worldwide, Fe deficiency anemia is recognized as the world’s most prevalent nutritional deficiency [1]. Infections, malnutrition, and Fe deficient diets are the main factors involved in its etiology [2]. Dietary Fe is present as heme Fe, obtained principally by the consumption of myoglobin and hemoglobin contained in meats and other animal products; and as non heme Fe, found in plants [3]. Both forms of Fe differ in their chemical form, uptake mechanisms, and absorption processes [4,5,6,7,8]. Heme Fe possesses higher bioavailability than non heme Fe [9,10]. The uptake mechanisms and factors that affect non heme Fe absorption have been studied extensively [4,11]. Research has shown that animal derived proteins enhance the absorption of non heme Fe [12,13,14,15], while legume proteins from soybeans [16,17,18], and other plant proteins decrease non heme Fe absorption [19,20,21]. Studies have shown increases in heme Fe absorption in the presence of animal proteins and hemoglobin [22,23,24,25], relative to the types of amino acids present [26], and the combination of hemoglobin with trypsin [27]. However, studies showing how plant proteins affect heme Fe absorption are scarce. The effect that plant proteins have on heme Fe absorption is controversial. Some authors have reported that soy proteins may increase heme Fe absorption [28], and others have described that the addition of soy protein products to a meal does not affect the total amount of heme Fe absorbed [29]. However, when soy proteins are used to partially substitute meat proteins, there is a decrease in both heme and non heme Fe absorption [17]. According to results previously reported by our group using Caco-2 cell models [30], heme Fe uptake decreases in the presence of all legume proteins extracted from peas, lentils, and soybeans. Purified zein protein produced an increase in heme Fe uptake, glutelin had no significant effect, and gliadin significantly decreased heme Fe uptake. The aim of this study is to determine the role of plant proteins extracted from cereals and legumes on heme Fe absorption in humans.

## 2. Experimental Section

### 2.1. Ethics

This study was approved by the Ethics Committee at the Institute of Nutrition and Food Technology, University of Chile. Radioisotope doses were approved by the Chilean Commission of Nuclear Energy. Each participant was informed about the benefits and risks of the study through an informed consent process. Participation was voluntary and all subjects were free to withdraw at any stage of the study. Procedures were conducted only after reading and signing the written informed consent form.

### 2.2. Heme Fe Labeled with Radioactive Isotopes

Fe isotopes of high specific activity (^59^Fe and ^55^Fe) (NEN Life Science Products, Inc., Boston, MA, USA) were used as intrinsic markers of heme Fe, which were injected into the marginal ear vein of five New Zealand rabbits (37 MBq ^59^Fe diluted in 0.1 mL of a solution of 9 g NaCl/L), and into the jugular veins of two Holstein Friesian calves (740 MBq ^55^Fe diluted in 3 mL of a solution of 9 g NaCl/L) [31]. Fifteen days after the injection of the isotopes, the rabbits and calves were sacrificed with an anesthetic overdose (10% thiopental at 25 mg/kg I.V.), and there exsanguinations were performed by jugular route [32]. The blood of the rabbits and calves were received in containers with 0.11 M sodium citrate at 9:1 ratio (v/v) citrate:blood and transferred immediately to the laboratory for processing. Heme Fe compounds were prepared using rabbit and calf blood.

### 2.3. Heme Fe Preparation

The collected blood was centrifuged at 3207× *g* for 10 min at 10 °C in a refrigerated centrifuge (RC3B Sorvall, Thermo Fisher Scientific, Waltham, MA, USA). Plasma and leukocytes were discarded and the red blood cells were washed three times with 9 g/L NaCl. Heme was extracted with the technique described by Labbé and Nishida [33]. Red blood cells from bovine and rabbits were treated with strontium 2% chloride in acetic acid and acetone solution (1:3), and were heated to boiling point to separate hemin from globin and other proteins. The final solution was filtered to eliminate protein residues, and heated again for about 1–2 h under an extraction hood to evaporate acetone and part of the water present in the mixture. The heme started to precipitate when the solution was at room temperature. The final product was washed with an acetic acid water solution, 1:1 ratio of ethanol and diethyl ether, then dried at 37 °C overnight. Labeled purified heme with a specific activity of 1913 kBq ^59^Fe (bovine source) and 274 kBq (rabbit source) ^55^Fe/mg of Fe was obtained. The labeled heme was mixed in dry form with dry untagged heme (obtained from three exsanguinated lambs) such that the result was a dose of 37 kBq ^59^Fe or 111 kBq ^55^Fe per 5 mg elemental Fe.

### 2.4. Protein

Pea and lentil protein isolates were processed according to the method described by Swanson [34] and rice protein was isolated using the method of Shih and Daigle [35]. Flour obtained from rice, peas and lentils was suspended in distilled water (20% w/v), and pH adjusted to 11 for rice and 10 for pea and lentil suspensions using 1 N NaOH. Next, insoluble compounds were separated by centrifugation, while the supernatants were adjusted to isoelectric points for each preparation (4.5 pH for pea and lentil, and 4.8 for rice) with 2 N HCl prior to centrifugation. Lastly, precipitates were lyophilized. Soy protein concentrate (Solae Company, Santiago, Chile) imported from DuPont™ Danisco^®^, USA, was used. The chemical composition was: moisture (max 8.0%), protein, dry basis (min 67%), ether extract (1.5%), and ash (8.0%). Proteins as isolates; zein from corn (Zein, Sigma Z3625, St. Louis, MO, USA) and gliadin from wheat (Gliadin, Sigma G3375, USA), were used.

The different types of proteins used in powder form were weighed and then 100 mL of distilled water was added to provide a solution to be consumed by subjects.

### 2.5. Study Design

Thirty healthy women (35 to 45 years old) participated in two experimental studies (15 per study). The participants did not receive any medication, vitamin, or mineral supplements six months prior to and during the study. The participants were not pregnant (confirmed by a negative human gonadotropin chorionic urine test) and were using a contraceptive method. Fasting subjects were enlisted into two protocols of Fe absorption.

#### 2.5.1. Study I. Effect of Proteins from Cereals on Heme Fe Absorption

Day 1: subjects ingested 5 mg of heme labeled with 3 μCi of ^55^Fe.

Day 2: subjects ingested 5 mg of heme labeled with 1 μCi of ^59^Fe plus 1.7 g of zein.

Day 14: an antecubital venous sample of 30 mL was used to determine Fe absorption. Then the participants ingested 5 mg of heme labeled with 3 μCi of ^55^Fe plus 1.7 g of gliadin.

Day 15: subjects ingested 5 mg of heme labeled with 1 μCi of ^59^Fe plus 1.7 g of glutelin isolate (also called oryzanin) from rice.

Day 28: A sample of 20 mL of blood was taken to determine Fe absorption.

#### 2.5.2. Study II. Effect of Proteins from Legumes on Heme Fe Absorption

Day 1: Subjects ingested 5 mg of heme labeled with 3 μCi of ^55^Fe.

Day 2: Subjects ingested 5 mg of heme labeled with 1 μCi of ^59^Fe plus concentrated soy protein.

Day 14: A sample of 30 mL of blood was taken to determine Fe absorption. Subjects then ingested 5 mg of heme labeled with 3 μCi of ^55^Fe plus isolate pea protein.

Day 15: Subjects ingested 5 mg of heme labeled with 1 μCi of ^59^Fe plus isolate lentil protein.

Day 28: A sample of 20 mL of blood was taken to determine Fe absorption.

### 2.6. Fe Status

Fe status was determined at baseline by the following biomarkers: hemoglobin (Hb) and mean corpuscular volume (MCV) (Coulter Model ZBI, Hialeah, Fla., and CELL-DYN 3200, ABBOTT Diagnostics, Abbott Park, IL, USA), free erythrocyte protoporphyrin (FEP) (Hematofluorimeter model 206D, AVIV, Lakewood, NJ, USA), serum Fe (SFe), total Fe binding capacity (TIBC), and serum ferritin (SF) by ELISA [36]. The percentage of transferrin saturation (TS) was calculated.

The definition of Fe status for each biomarker, with the exception of SF, was based on cut-off points proposed by the Centers for Disease Control and Prevention [37]. Depleted Fe stores were defined as SF < 15 μg/L [38], Fe deficiency without anemia was defined as normal Hb and two or more altered biomarkers (MCV, FEP, SFe, TIBC, TS or SF), and Fe deficiency anemia was defined as below-normal Hb and two or more of any of the previously mentioned altered biomarkers.

### 2.7. Fe Absorption

The calculation of total radioactivity in blood samples was performed by the double isotope labeling technique, described by Eakins and Brown [39], in a liquid scintillation counter (Packard Canberra Company TRI-CAB 1600 TR). The counts were conducted with two blank samples, quadruplicate of the aliquots ingested by the subjects of ^55^Fe and ^59^Fe as standards, and sextuplicates of samples from each subject. The percentages of absorption were calculated on the basis of blood volumes estimated from height and weight [40], assuming 80% incorporation of the radioisotope into the erythrocyte [41].

### 2.8. Statistical Analysis

A sample size of nine subjects was calculated for each study, using the software PRIMER, version 3.02 (PRIMER-E Ltd., Plymouth, UK), option “power and sample size ANOVA”. The sample size was calculated with an alpha of 0.05, a power of 80%, an expected residual standard deviation of three, a number of treatment groups of four and a minimum detectable difference of 5% in the absorption of heme Fe. For each study, 15 volunteers were considered in order to account for the withdrawal of subjects due to the rejection of intake, and/or the presence of diarrhea or vomiting, producing significant losses of the administered compounds.

Continuous variables were retransformed into antilogarithms to recover the original units and were expressed as geometric means and range ±1 SD (standard deviation). Intra-group statistical comparisons were performed with each subject as their own control. Pearson coefficients were used to correlate SF and heme Fe absorption. ANOVA for repeated measures; *post hoc* Dunnett’s and student *T*-test were applied using the statistical software STATA 11.0 (Stata Corp LP, College Station, TX, USA). A *p*-value < 0.05 was considered as significant.

## 3. Results

### 3.1. Baseline Characteristics of Participants

The average age was 36 ± 3 and 39 ± 5 years old in studies I and II, respectively. There were no differences in Fe status biomarkers across subjects who participated in studies I and II. The following *p*-values were obtained from student *T* tests for Hb, MCV, FEP, SFe, TIBC, TS and SF biomarkers: 0.77, 1.00, 0.61, 0.21, 0.56, 0.19, and 0.08, respectively. In study I, 9 participants had normal Fe status, 1 depleted Fe stores, 4 Fe deficiency without anemia, and 1 presented Fe deficiency anemia (Table 1). In study II, 13 women had normal Fe status, 1 depleted Fe stores and 1 woman was Fe deficient (Table 2).

**Table 1:** Iron status biomarkers of the subjects in study I.

Subjects	Hb (g/L)	MCV (fL)	FEP (μg/dL)	SFe (μmol/L)	TIBC (μmol/L)	TS (%)	SF (μg/L)
1	139	86	43	69	310	22	24
2	135	89	37	60	337	18	8
3	141	88	57	67	332	20	29
4	138	93	51	124	267	47	47
5	121	88	54	52	321	16	9
6	138	93	69	74	299	25	18
7	138	86	52	50	335	15	22
8	140	90	60	89	307	29	24
9	135	87	51	42	318	13	13
10	141	89	51	89	313	29	66
11	126	92	57	58	457	13	8
12	144	94	63	45	288	16	15
13	152	95	49	116	294	40	51
14	155	88	43	30	348	9	23
15	114	80	83	29	348	8	5
mean ± SD	137 ± 11	89 ± 4	55 ± 11	66 ± 28	325 ± 43	18 (11–31) *^a^*	19 (9–39) *^a^*
Cut-off points *^b^*	<120	<80	>70	<60	>450	<15	<15 *^c^*

Hb, Hemoglobin; MCV, median corpuscular volume; FEP, free erythrocyte protoporphyrin; SFe, serum Fe; TIBC, total Fe binding capacity; TS, transferrin saturation; SF, serum ferritin. *^a^* Values are expressed as geometric means and ranges ±1 SD. *^b^* Cut-off points to define iron status [37]. *^c^* Cut-off points to define SF [38].

**Table 2:** Iron status biomarkers of the subjects in study II.

Subjects	Hb (g/L)	MCV (fL)	FEP (μg/dL)	SFe (μmol/L)	TIBC (μmol/L)	TS (%)	SF (μg/L)
1	150	95	69	100	339	30	28
2	131	88	54	35	241	15	41
3	134	88	57	67	342	20	17
4	126	92	63	109	283	38	21
5	140	91	66	72	381	19	9
6	140	90	66	58	356	16	25
7	140	83	46	33	260	13	36
8	141	90	69	69	291	24	32
9	142	82	49	76	314	24	19
10	126	89	51	69	283	24	40
11	133	90	40	68	288	24	56
12	137	88	51	100	347	29	55
13	143	92	71	63	300	21	22
14	136	88	49	111	314	35	21
15	146	94	49	90	300	30	21
mean ± SD *^a^*	138 ± 7	89 ± 4	57 ± 10	75 ± 24	309 ± 38	23 (17–32) *^a^*	27 (16–43) *^a^*
Cut-off points *^b^*	<120	<80	>70	<60	>450	<15	<15 *^c^*

Hb, Hemoglobin; MCV, median corpuscular volume; FEP, free erythrocyte protoporphyrin; SFe, serum Fe; TIBC, total Fe binding capacity; TS, transferrin saturation; SF, serum ferritin. *^a^* Values are expressed as geometric means and ranges ±1 SD. *^b^* Cut-off points to define iron status [37]. *^c^* Cut-off points to define SF [38].

### 3.2. Heme Fe Absorption Studies

The absorption of heme Fe from heme alone and heme plus cereal protein are shown in Table 3 (study I). Heme Fe absorption at baseline was 6.2%, and with the addition zein and gliadin proteins, a slight increase was observed, however this was not significant. Fe absorption of heme plus glutelin was less than heme only, however this was also not significant.

**Table 3:** Heme iron absorption of heme only and heme plus cereal proteins (zein, gliadin, glutelin).

Subjects	Heme Fe Absorption (%)				Ratios		
	^55^Heme (H)	^59^Heme + zein (A)	^55^Heme + gliadin (B)	^59^Heme + glutelin (C)	A/H	B/H	C/H
1	12.2	13.2	11.4	9.3	1.08	0.93	0.77
2	9.6	7.5	7.7	6.0	0.78	0.80	0.62
3	4.2	4.1	5.8	5.2	0.97	1.37	1.22
4	1.6	1.6	4.0	0.9	0.99	2.41	0.53
5	7.8	9.6	7.5	3.9	1.24	0.96	0.50
6	11.5	16.8	14.1	14.2	1.46	1.23	1.24
7	6.8	9.1	7.5	4.6	1.35	1.10	0.68
8	3.8	5.1	5.6	5.1	1.37	1.50	1.37
9	8.5	7.4	12.1	8.2	0.87	1.42	0.96
10	3.7	3.6	3.2	3.4	0.96	0.85	0.91
11	12.7	15.2	8.7	10.9	1.20	0.68	0.86
12	8.2	9.5	12.8	10.1	1.16	1.56	1.23
13	2.3	3.5	4.3	4.0	1.52	1.88	1.74
14	6.0	7.7	8.2	7.1	1.28	1.37	1.18
15	9.7	14.1	10.6	12.8	1.46	1.10	1.32
Mean *^a^*	6.2	7.2	7.5	5.9	1.16	1.21	0.95
SD *^a^*	3.3–11.6	3.7–13.8	4.8–11.8	2.9–11.8	0.94–1.42	0.86–1.70	0.65–1.38
SS *^b^*		N.S	N.S	N.S			
r *^c^*	−0.76	−0.73	−0.68	−0.60			

*^a^* Geometric mean and range ±1 SD. *^b^* Statistical significance (SS) calculated with respect to heme absorption. N.S, non significant (*p* < 0.05). *^c^* Pearson correlation coefficients between serum ferritin and Fe absorption.

The absorption of heme Fe from heme alone and heme plus legume proteins are shown in Table 4 (study II). Soy protein concentrate showed a significant decrease on the absorption of heme Fe. Soybean proteins decreased heme Fe absorption by 34%. Pea and lentil protein concentrates showed no significant effect on heme Fe absorption.

**Table 4:** Heme iron absorption of heme only and heme plus legume protein (soy, pea, lentil).

Subjects	Heme Iron Absorption (%)				Ratios		
	^55^Heme (H)	^59^Heme + soy (A)	^55^Heme + pea (B)	^59^Heme + lentil (C)	A/H	B/H	C/H
1	24.0	6.6	9.3	14.0	0.28	0.39	0.58
2	8.4	8.5	16.0	24.8	1.01	1.91	2.94
3	12.9	10.6	10.1	13.8	0.82	0.79	1.07
4	13.7	11.4	16.4	20.2	0.84	1.20	1.48
5	18.9	9.2	11.4	19.1	0.49	0.61	1.01
6	12.3	7.9	9.3	10.6	0.64	0.76	0.86
7	9.4	5.6	2.9	3.1	0.59	0.31	0.33
8	9.5	4.3	5.8	5.0	0.46	0.62	0.53
9	13.7	6.0	10.8	9.5	0.44	0.78	0.69
10	9.0	6.4	5.6	6.4	0.71	0.63	0.71
11	12.7	5.2	7.3	9.1	0.41	0.57	0.71
12	6.7	8.8	4.9	3.8	1.32	0.73	0.57
13	10.0	15.9	11.3	11.2	1.59	1.13	1.12
14	8.6	4.1	7.9	8.3	0.47	0.91	0.97
15	6.5	6.4	5.1	3.9	0.98	0.80	0.60
Mean *^a^*	11.0	7.3	8.1	9.1	0.66	0.74	0.82
SD *^a^*	7.7–15.8	5.0–10.6	5.1–13.0	4.8–17.2	0.41–1.07	0.47–1.15	0.49–1.37
SS *^b^*		*p* < 0.02	N.S	N.S			
r *^c^*	−0.44	−0.29	−0.42	−0.41			

*^a^* Geometric mean and range ±1 SD. *^b^* Statistical significance (SS) calculated with respect to heme absorption. N.S: non significant. *^c^* Pearson correlation coefficients between serum ferritin and Fe absorption.

As expected, an inverse relationship between SF and Fe absorption was observed in both studies (Table 3 and Table 4), but in study I, these correlations were higher and significant (*p* < 0.01) compared with study II (*p* < 0.458). It is possible that subjects who participated in study I tended to have lower Fe stores, but these differences were not significant compared with subjects from study II. However, they did present a higher range of SF.

**Figure 1:**
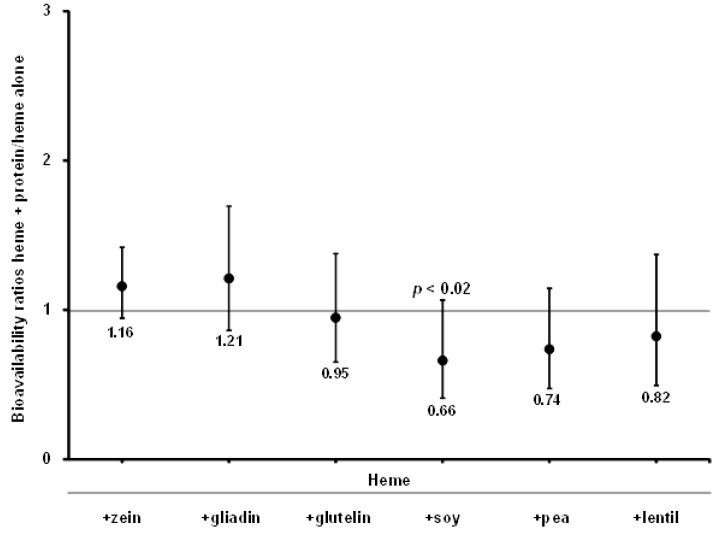
Ratios between heme Fe absorption with proteins and heme Fe absorption alone. Bars represent ±1 SD.

The absorption ratios of “heme + proteins:heme alone”, for both studies I and II, indicate that plant proteins did not promote the absorption of heme Fe. In fact, legume proteins from soybeans (*p* < 0.02) decreased heme Fe absorption (Figure 1).

## 4. Discussion

The absorption of heme Fe was low in both studies, especially in study I, although this group contained a greater number of individuals with different Fe deficiency states, who are able to absorb higher amounts of Fe from the diet [4,14,24]. Subjects 4 and 13 from study I absorbed very low amounts of heme Fe (1.6% and 2.3%, respectively) (Table 3), in contrast with some subjects from study II who absorbed higher amounts of heme Fe (24.0% and 18.9%) (Table 4). Heme Fe absorption not only depends on the reserves of Fe, but on other factors such as intestinal motility, inflammation/infection, hypoxia, erythropoiesis rate, among others [4,5]. The variability of heme Fe absorption in studies I and II was found to be within what can be expected for Fe absorption studies conducted on a sample population with different Fe status [6]. To compensate for this variability, there were more than 9 subjects (the recommended number of individuals for radioisotope-labeled Fe studies) in studies I and II, which were considered as controls [6,24]. With respect to the low heme Fe absorption rates in both studies, we suggest that it may be related to the fact that heme Fe (without globin) used to feed the participants is poorly absorbed when consumed as a prosthetic group [22,24]. Our research group has recently reported that proteins from animal source foods and their digestion products do not enhance heme Fe absorption, suggesting that the erythrocyte stroma could be one of the factors that increases heme Fe absorption [42]. In the present study we did not observe a significant effect on heme Fe absorption by cereal proteins. In the case of legumes, soy exerted an inhibitory effect. There is a consensus among researchers that soybeans and soy products are relatively poor sources of available Fe and decrease non heme Fe absorption [16,18]. However, their effect on the absorption of heme Fe has been poorly investigated and there are contradictory results. The results of this study are not consistent with what we previously reported in Caco-2 cells [29], due to several biological factors that affect the Fe absorption in human. Hallberg and Rossander [17] found that when half of the meat was replaced by “soy-meat” in hamburgers, the total amount of Fe absorbed, including heme Fe, was much lower. The addition of heme Fe as blood to the half meat, half soy hamburgers resulted in an increase in the absorption of Fe to the level measured in all meat hamburgers.

In this study, heme Fe was separated from erythrocyte membranes. It is important to highlight that we used purified heme without globin. Lynch *et al.* [28] used 3 μCi of a ^55^Fe hemoglobin solution isolated from a pathogen-free rabbit as heme Fe. This could account for the enhancing effect of soy proteins on the absorption of heme Fe described by Lynch *et al.* [28], since it has been postulated that globin could improve heme Fe absorption [22,24].

It is crucial to define the effect of soy proteins on the absorption of heme Fe, because of its widespread use as a binder for improving yields, a gelling agent to enhance emulsion stability, a cheap meat replacement, and as a “meat extender” in hamburgers, meatballs, and similar meat products [43,44]. According to this study and to others by authors such as Hallberg and Rossander [17] and Villarroel *et al.* [29] soy proteins decrease heme Fe absorption. Further research is needed to elucidate why soy protein concentrate decreases the absorption of heme Fe.

Cereal proteins, such as zein, gliadin, and glutelin and the legume proteins from peas and lentils, displayed no effect on the absorption of heme Fe. The absence of inhibition can be interpreted as beneficial because cereals and legumes are key sources of proteins, and most rural diets are predominantly based on cereals, legumes, or starchy roots and tubers in developing countries [45]. A high prevalence of Fe deficiency has been reported in developing countries. This can be attributed to the lack or low availability of animal source foods, and also to the effect of plant Fe absorption inhibitors such as phytate, oxalic acid and polyphenols which inhibit non heme Fe absorption by forming insoluble complexes in the intestine [46,47].

Fe deficiency anemia continues to be a major micronutrient deficiency disorder in the world [1]. Considerable efforts have been made to eliminate or reduce the prevalence of Fe deficiency anemia. Strategies to reduce this disease include (1) Fe supplementation, (2) Fe fortification, and (3) dietary diversification [48]. In all of these strategies heme Fe has gained great importance due to its high bioavailability (two to three times more than non heme Fe), its resistance to becoming affected by other dietary factors [49], and due to its low ability for causing gastrointestinal side effects [50]. Consequently, several authors have used heme Fe as a supplement/fortifier with positive results [51,52]. It is important to further explore the role of potential dietary factors that may increase or decrease heme Fe absorption in order to improve the strategies available to combat Fe deficiency.

## 5. Conclusions

In summary, plant derived proteins from cereals did not affect heme Fe absorption. In the case of legumes, proteins from soybeans exerted an inhibitory effect. The reported effects of plant proteins are only relevant to purified heme Fe and not to dietary sources of hemoglobin.
